# A multi-center study on glucometabolic response to bariatric surgery for different subtypes of obesity

**DOI:** 10.3389/fendo.2022.989202

**Published:** 2022-11-03

**Authors:** Yao Liu, Chunjun Sheng, Wenhuan Feng, Fang Sun, Jingjing Zhang, Ying Chen, Lili Su, Jia Liu, Lei Du, Xuyang Jia, Hui You, Xiu Huang, Shandong Wu, Ziwei Lin, Shen Qu

**Affiliations:** ^1^ Department of Endocrinology and Metabolism, Shanghai Tenth People’s Hospital, Tongji University School of Medicine, Shanghai, China; ^2^ Department of Endocrinology, Drum Tower Hospital Affiliated to Nanjing University Medical School, Nanjing, China; ^3^ Department of Hypertension and Endocrinology, Daping Hospital, Army Medical University, Chongqing Institute of Hypertension, Chongqing, China; ^4^ National Clinical Research Center for Metabolic Diseases, Metabolic Syndrome Research Center, Key Laboratory of Diabetes Immunology, Ministry of Education, The Second Xiangya Hospital of Central South University, Changsha, Hunan, China; ^5^ Department of Metabolism and Endocrinology, The Second Xiangya Hospital of Central South University, Changsha, Hunan, China; ^6^ Ministry of Education, Key Laboratory of Metabolism and Molecular Medicine, Department of Endocrinology and Metabolism, Zhongshan Hospital, Fudan University, Shanghai, China; ^7^ Department of Radiology, Department of Biomedical Informatics, Department of Bioengineering, Intelligent Systems Program, University of Pittsburgh, Pittsburgh, PA, United States; ^8^ Department of Biomedical Informatics, Intelligent Systems Program, University of Pittsburgh, Pittsburgh, PA, United States; ^9^ Department of Bioengineering, Intelligent Systems Program, University of Pittsburgh, Pittsburgh, PA, United States

**Keywords:** obesity subtypes, bariatric surgery, diabetes, hyperinsulinemia, hypoglycemia

## Abstract

**Objectives:**

To assess the benefit of a bariatric surgery in four artificial intelligence-identified metabolic (AIM) subtypes of obesity with respect to the improvement of glucometabolism and the remission of diabetes and hyperinsulinemia.

**Methods:**

This multicenter retrospective study prospectively collected data from five hospitals in China from 2010 to 2021. At baseline 1008 patients who underwent a bariatric surgery were enrolled (median age 31 years; median BMI 38.1kg/m^2^; 57.40% women) and grouped into the four AIM subtypes. Baseline and follow-up data (506 and 359 patients at 3- and 12-month post-surgery) were collected for longitudinal effect analysis.

**Results:**

Out of the four AIM subgroups, hypometabolic obesity (LMO) group was characterized by decompensated insulin secretion and high incidence of diabetes (99.2%) pre-surgery. After surgery, 62.1% of LMO patients with diabetes achieved remission, lower than the other three subgroups. Still, the bariatric surgery significantly reduced their blood glucose (median HbA1c decreased by 27.2%). The hypermetabolic obesity-hyperinsulinemia (HMO-I) group was characterized by severe insulin resistance and high incidence of hyperinsulinemia (87.8%) pre-surgery, which had been greatly alleviated post-surgery. For both metabolic healthy obesity (MHO) and hypermetabolic obesity-hyperuricemia (HMO-U) groups who showed a relatively healthy glucometabolism pre-surgery, rate of glucometabolic comorbidities improved moderately post-surgery.

**Conclusion:**

In terms of glucometabolism, the four AIM subtypes of patients benefited differently from a bariatric surgery, which significantly relieved hyperglycemia and hyperinsulinemia for the LMO and HMO-I patients, respectively. The AIM-based subtypes may help better inform clinical decisions on bariatric surgery and patient counseling pertaining to post-surgery outcomes.

## Introduction

Bariatric surgery has been recommended as one of the standard treatments for obese patients with type 2 diabetes (T2DM) by American Diabetes Associations (ADA) since 2009 (1). It outperforms traditional lifestyle interventions and medical treatments in glycemic control and insulin resistance relief (2–5). Although surgery indications (e.g., criteria for BMI, age, disease duration, C-peptide level, etc.) have been applied in patient’s selection (6), surgery effects still vary remarkably between individuals. Previous studies have reported the average postoperative remission rate of diabetes, ranging from 30% to 95% (3–5, 7, 8), as well as the percentage decrease of insulin resistance [indicated by homeostatic model assessment of insulin resistance (HOMA-IR)], ranging from 12 to 55% (9). This may relate to the heterogeneity of the disease in terms of clinical presentation and pathogenesis. Towards precision treatment, a more refined metabolic classification of obesity is highly demanded, aiming to identify the patients who may benefit more from a bariatric surgery.

In recent years, artificial intelligence techniques are under intensive investigation and evaluation in medicine. Data-driven machine learning modeling provides an intelligent method (10) to mine up large and multi-dimensional medical data for refined classification, quantitative analysis, and patient outcome prediction. In a previous study (11), we identified a novel classification of four metabolic subtypes of obesity by using unsupervised machine learning. For self-completeness, here is a brief summary of the four subtypes: i) metabolic healthy obesity (MHO), characterized by a relatively healthy-metabolic status with the lowest incidences of comorbidities; ii) hypermetabolic obesity-hyperuricemia (HMO-U), characterized by high uric acid but still relatively healthy glucometabolism; iii) hypermetabolic obesity-hyperinsulinemia (HMO-I), distinguished by overcompensated insulin secretion and hypoglycemia; and iv) hypometabolic obesity (LMO), characterized by decompensated insulin secretion, high glucose, and the worst glucolipid metabolism. We named them in short as artificial intelligence-identified metabolic (AIM) subtypes of obesity.

In view that the four AIM subtypes showed different glucometabolism at baseline, we hypothesized that they may benefit differently from a bariatric surgery in terms of diabetes and hyperinsulinemia remission. The goal of this study is to test this hypothesis by conducting a multicenter study to examine the longitudinal effects of bariatric surgery at 3- and 12-month post-surgery with respect to the four AIM subtypes.

## Material and methods

### Study population

We conducted a multicenter retrospective study with prospectively collected data for patients from five different hospitals in China (ClinicalTrials No. NCT04282837), which was approved by a local ethics committee and the institutional review boards of the participating institutions. Patients who underwent their first-time bariatric surgeries were included; here, those were the patients with a BMI ≥ 32.5 kg/m^2^ or a BMI 27.5 to 32.5 kg/m^2^ with one or more severe obesity-related complications, according to the AACE/TOS/ASMBS/OMA/ASA 2019 Guidelines (6) adjusted for Chinese populations. Patients were excluded if they: (1) had taken exogenous insulin or medication (which may have affected glucometabolism) in the recent three months before the surgery; (2) were diagnosed with type 1 diabetes, secondary diabetes, hereditary disease, or severe disease (e.g., malignant tumor, heart failure, liver failure, Prader-Willi syndrome, Bardet-Biedl syndrome, Alström syndrome etc.); (3) were in the gestation or lactation; or (4) had substantial clinical data missing and were excluded in the first step of the AIM subtyping.

In this multi-center database, a total of 1223 patients were documented according to the inclusion criteria; 215 patients were excluded according to the exclusion criteria. At the end, this study enrolled a total of 1008 Chinese patients with obesity (median age 31 years; median body mass index (BMI) 38.1 kg/m^2^; 57.40% women) presented during January 2010 to October 2021 from five hospitals: Shanghai Tenth People’s Hospital (Cohort-1, n = 468), Nanjing Drum Tower Hospital (Cohort-2, n = 262), Shanghai Zhongshan Hospital (Cohort-3, n = 81), Chongqing Daping Hospital (Cohort-4, n = 84), and Changsha second Xiangya Hospital (Cohort-5, n = 113). Among all the enrolled patients, 79.4% underwent laparoscopic sleeve gastrectomy and 20.6% underwent Roux-en-Y gastric bypass. After surgery, 506 patients and 359 patients were performed with follow-up visits at 3- and 12-month, respectively. In addition, 102 normal-weight (NW) healthy subjects (with normal glucose tolerance and normal uric acid) from Shanghai Tenth People’s Hospital (median age 31 years; median BMI 21.7 kg/m^2^; 58.80% women) were included as controls for statistical analysis. These NW healthy participants presented to the clinic for routine health examinations.

### Measurements and calculations

A local multidisciplinary team assessed and managed patients through preoperative and postoperative procedures. BMI was calculated as weight (kg)/height^2^ (m^2^). Waist/hip ratio (WHR) was calculated as waist circumference (WC, cm)/hip circumference (HC, cm). Percentage excess weight loss (%EWL) was calculated as (baseline weight – last weight)/(baseline weight – healthy weight) × 100%.The healthy weight was defined as BMI = 24 kg/m^2^, according to the Chinese criteria (12).

The area under the curve (AUC) of glucose (glucose_AUC_) and insulin (insulin_AUC_) was calculated using the trapezoidal rule at four data points of 0, 30, 60, and 120 min during oral glucose tolerance test (OGTT, 75g glucose). For patients in Cohort-5, C-peptide instead of insulin was routinely measured during OGTT. Thus, a linear regression model was established using C-peptide to estimate insulin_AUC_ during OGTT (see **Supplementary Material**).

Pancreatic β-cell function was estimated using the insulinogenic index (IGI) (13) and HOMA of β-cell function (HOMA-β) (14). IGI was calculated as △insulin_0-30min_/△glucose_0-30min_. HOMA-β was calculated as 20 × insulin_0min/_(glucose_0min_ − 3.5). Insulin resistance was determined by HOMA-IR (14) as glucose_0min_ × insulin_0min_/22.5. Insulin sensitivity was measured by whole-body insulin sensitivity index (WBISI) (15) as 10000/square root of [(glucose_0min_ × 18 × insulin_0min_) × (glucose_mean_ × 18 × insulin_mean_)], and mean glucose or insulin during OGTT were calculated as the average of measurements at 0, 30, 60, and 120 min. Disposition indices (DI) were used to estimate relative insulin secretion compared to insulin resistance or sensitivity and were calculated as HOMA-β/HOMA-IR and IGI × WBISI. For all the formulas above, glucose and insulin were calculated in mmol/L and mU/L, respectively.

### Definitions

Successful weight loss was defined as %EWL > 50% at 12 months (16).

Diabetes was defined according to the ADA guidelines (17): Glucose_0min_ ≥ 7.0 mmol/L and/or glucose_120min_ ≥ 11.1 mmol/L during OGTT and/or HbA1C ≥ 6.5% and/or confirmed diabetes. In addition, according to ADA criteria (18), complete remission from diabetes was defined as glucose_0min_ ≤ 5.6 mmol/L and HbA1c ≤ 6.0% in the absence of active pharmacological treatment, while partial remission was defined as glucose_0min_ 5.6–6.9 mmol/L and HbA1c 6.0–6.5% in the absence of active pharmacological treatment.

Hyperglycemia was defined as glucose_0min_ ≥ 6.1 mmol/L and/or glucose_120min_ ≥ 7.8 mmol/L during OGTT and/or HbA1C ≥ 6.5% and/or confirmed diabetes.

Hyperinsulinemia was defined as insulin_0min_ ≥ 24 mU/L.

Hypoglycemia was defined as glucose < 2.8 mmol/L at any time during OGTT (0, 30, 60, 120, and 180 min) (19).

### AIM grouping of patients with obesity

The modeling method for AIM subgrouping has been described in our previous study (11) (brief summary see **Supplementary Material**). In the present study, we assigned the patients in the new cohort to the prior established clusters of the AIM subgrouping models in the previous study (11), according to the similarity of a patient’s characteristics to each cluster of the four AIM subgroups. This similarity was measured by the Euclidian distance to the nearest cluster’s center of a model. Clustering was implemented by SPSS version 18 (IBM, Chicago, IL, USA), and all variables were normalized (mean value of 0 and standard deviation (SD) of 1) before cluster analyses.

### Statistical analysis

The clinical implications of variables related to glucometabolism and morbidity were compared within the four obesity subgroups. Continuous variables were expressed as the median (interquartile range: 25–75%) since most were skewed distribution, while variables not normally distributed were logarithmically or square root transformed before statistical analysis, conducted using SPSS version 26 (IBM, Chicago, IL, USA). ANOVA or ANCOVA was used for assessing the differences among continuous variables as appropriate. Bonferroni correction was used for the *post hoc* analysis. Differences in ratio variables were assessed by the chi-square test. For all analyses, P-values were two-tailed, with P < 0.05 indicating statistical significance.

## Results

### The four AIM subtypes of obesity showed different effects of weight loss after bariatric surgery

At baseline, 1008 patients were included for analysis and AIM subgrouping, leading to 337, 327, 42, and 262 patients categorized into MHO, HMO-U, HMO-I, and LMO, respectively. For the subset of 506 patients with 3-month follow-up, the distribution of MHO, HMO-U, HMO-I, and LMO is 171, 172, 23, and 140 patients, respectively. Likewise, for the 359 patients with 12-month follow-up, the distribution of the four subtypes is 118, 111, 14, and 116 patients, respectively.

**Table 1** shows the anthropometry characteristics of each AIM subtype of obesity patients at baseline and 12-month post-surgery. Patients in the HMO-U and HMO-I subgroups were characterized by higher pre-surgery weight and BMI, consistent with what was observed in our previous study (11). During the 12-month time period after surgery, patients in both the HMO-U and HMO-I subgroups showed the most weight loss (33.5% and 34.0%, respectively) and WC loss (23.9% and 26.7%, respectively), while patients in the LMO group showed the least weight loss (24.4%) and WC loss (18.2%). At 12-month post-surgery, 91.3%, 96.9%, 91.7%, and 90.4% of the patients in MHO, HMO-U, HMO-I, and LMO, respectively, achieved successful weight loss, and patients in all the four subgroups showed similar weight-related anthropometry characteristics at the end.

**Table 1:** The anthropometry characteristics of the four AIM subgroups of obesity patients at baseline and 12-month post-surgery.

	MHO	HMO-U	HMO-I	LMO	p value^&^
**N**	377	327	42	262	
**Man/Woman (woman %)**	150/227 (60.2%)	134/193 (59.0%)	25/17 (40.5%)	120/142 (54.2%)	0.055
**Age (years)**	31 (25,37)	28 (23,34)	27 (20,32)	37 (30,46)	<0.0001
**Obesity duration (years)**	10.0 (5.0,16.3)	10.0 (5.0,18.0)	10.0 (5.0,17.0)	15.0 (9.0,20.8)	<0.0001
**Type of surgery (RYGB%)**	17.0%	13.1%	9.5%	37.0%	<0.0001
**Rate of diabetes (%)**	33.2%	32.1%	9.5%	99.2%	<0.0001
**Diabetes duration (years)**	0.5 (0,2.5)	0 (0,1.0)	0 (0,0.1)	1.0 (0,5.0)	<0.0001
**Weight (kg)**					
Baseline	103.5 (90.0, 124.6)	113.4 (98.3, 130.0)	116.6 (101.7, 129.7)	100.0 (86.0, 120.1)	<0.0001
12 months	74.1 (64.1, 87.3) ^#^	78.4 (66.9, 89.1) ^#^	80.2 (71.1, 95.9) ^#^	71.7 (62.1, 83.2) ^#^	0.354
Absolute change	-31.6 (-41.8, -23.7)	-37.8 (-47.2, -31.3)	-37.4 (-53.8, -31.9)	-24.6 (-31.8, -17.1)	<0.0001
Percent change (%)	-29.4% (-35.7%, -25.0%)	-33.5% (-38.3%, -26.9%)	-34.0% (-37.6%, -25.0%)	-24.4% (-30.2%, -20.3%)	<0.0001
**BMI (kg/m^2^)**					
Baseline	37.2 (33.3, 41.6)	39.2 (35.4, 44.0)	39.2 (36.3, 41.8)	36.4 (31.5, 41.0)	<0.0001
12 months	26.9 (23.5, 30.1) ^#^	27.0 (24.5, 30.3) ^#^	27.1 (24.7, 29.1) ^#^	26.7 (23.3, 29.4) ^#^	0.781
Absolute change	-11.1 (-13.9, -9.2)	-13.3 (-16.3, -10.8)	-12.5 (-16.2, -9.2)	-8.9 (-11.3, -6.4)	<0.0001
Percent change (%)	-29.4% (-35.7%, -25.0%)	-33.5% (-38.3%, -26.9%)	-34.0% (-37.6%, -25.0%)	-24.4% (-30.2%, -20.3%)	<0.0001
**Excess weight (kg)**					
Baseline	37.9 (25.3, 52.3)	45.3 (31.8, 58.4)	43.1 (34.8, 55.2)	33.8 (21.1, 49.3)	<0.0001
12 months	8.1 (-1.3, 17.4) ^#^	8.2 (1.4, 17.0) ^#^	10.3 (1.9, 16.9) ^#^	7.2 (-2.1, 15.9) ^#^	0.696
Absolute change	-31.6 (-41.8, -23.7)	-37.8 (-47.2, -31.3)	-37.4 (-53.8, -31.9)	-24.6 (-31.8, -17.1)	<0.0001
Percent change (%)	-81.1% (-105.6%, -66.2%)	-83.1% (-96.6%, -68.7%)	-79.3% (-95.8%, -65.9%)	-77.7% (-111.8%, -62.0%)	0.756
**WC (cm)**					
Baseline	115 (106, 129)	120 (109, 130)	120 (111, 130)	116 (105, 128)	0.011
12 months	89 (82, 101) ^#^	92 (85, 98) ^#^	90 (84, 96) ^#^	92 (83, 98) ^#^	0.885
Absolute change	-27 (-35, -19)	-31 (-35, -23)	-30 (-46, -26)	-21 (-29, -14)	<0.0001
Percent change (%)	-22.8% (-28.6%, -16.6%)	-23.9% (-29.9%, -19.5%)	-26.7% (-30.9%, -22.0%)	-18.2% (-23.7%, -14.8%)	<0.0001
**HC (cm)**					
Baseline	120 (112, 129)	124 (114, 132)	120 (113, 132)	115 (107, 125)	<0.0001
12 months	101 (95, 106) ^#^	100 (94, 107) ^#^	103 (98, 107) ^#^	98 (91, 105) ^#^	0.486
Absolute change	-20 (-25, -13)	-23 (-28, -16)	-19 (-33, -11)	-13 (-19, -8)	<0.0001
Percent change (%)	-16.0% (-19.7%, -11.6%)	-18.3% (-21.6%, -12.7%)	-15.3% (-22.7%, -9.5%)	-11.6% (-15.0%, -7.7%)	<0.0001
**WHR (ratio)**					
Baseline	0.98 (0.91, 1.02)	0.97 (0.93, 1.03)	1.02 (0.96, 1.07)	1.00 (0.96, 1.04)	0.006
12 months	0.91 (0.85, 0.95) ^#^	0.90 (0.87, 0.95) ^#^	0.88 (0.83, 0.93) ^#^	0.92 (0.88, 0.97) ^#^	0.065
Absolute change	-0.08 (-0.12, -0.03)	-0.08 (-0.13, -0.04)	-0.13 (-0.18, -0.10)	-0.07 (-0.10, -0.04)	0.065
Percent change (%)	-8.2% (-11.8%, -3.7%)	-8.3% (-12.2%, -3.9%)	-12.8% (-16.8%, -10.8%)	-6.7% (-10.4%, -4.1%)	0.062

Values are shown as ratio or median (IQR 25-75%), and analysis were adjusted for sex and age (except analysis for sex and age). MHO, metabolic healthy obesity; HMO-U, hypermetabolic obesity-hyperuricemia subtype; HMO-I, hypermetabolic obesity-hyperinsulinemia subtype; LMO, hypometabolic obesity; BMI, body mass index; WC, waist circumference; HC, hip circumference; WHR, Waist-to-Hip Ratio. ^#^P< 0.01 vs. baseline; ^&^ the overall ANCOVA p values.

### The four AIM subgroups of obesity benefited differently on glucometabolism from bariatric surgery

**Figure 1:**
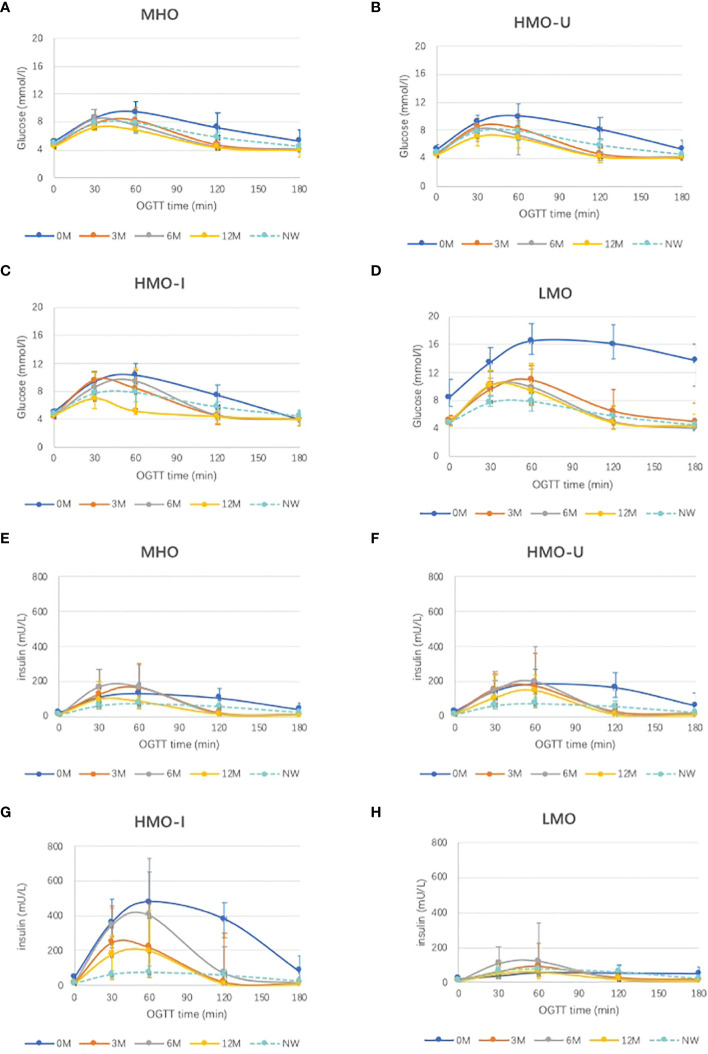
Comparison of different AIM subgroups of obesity and normal-weight controls for oral glucose tolerance test (OGTT) curves at baseline and post-surgery follow-ups. **(A-D)**, OGTT for glucose; **(E-H)**, OGTT for insulin. MHO, metabolic healthy obesity; HMO-I, hypermetabolic obesity hyperinsulinemia subtype; HMO-U, hypermetabolic obesity hyperuricemia subtype; LMO, hypometabolic obesity; NW, normal weight control; 0M, pre- surgery; 3M, 3-month post-surgery; 12M, 12-month post-surgery.

**Table 2** and **Figure 1** show the glucometabolism of the four AIM subgroups of obesity at baseline and follow-ups post-surgery.

**Table 2:** The glucometabolism of the four AIM subgroups of obesity and normal weight controls at baseline and 12-month post-surgery.

	MHO	HMO-U	HMO-I	LMO	NW	P value^&^
**Fasting glucose (mmol/L)**						
Baseline	5.10 (4.70, 5.60)	5.20 (4.76, 5.90)	5.05 (4.50, 5.49)	8.50 (7.10, 11.00)		<0.0001
12 months	4.47 (4.10, 4.76) *^#^	4.40 (4.15, 4.70) *^#^	4.60 (4.40, 4.91) ^#^	4.87 (4.38, 5.70) ^#^	4.86 (4.60, 5.22)	<0.0001
Absolute change	-0.63 (-0.27, -1.35)	-0.80 (-0.30, -1.50)	-0.60 (0.15, -1.12)	-3.46 (-1.70, -5.56)		<0.0001
Percent change (%)	-13.3% (-23.5%, -5.9%)	-15.3% (-25.9%, -6.4%)	-12.0% (-19.4%, 3.4%)	-40.9% (-53.6%, -27.4%)		<0.0001
**OGTT 2h glucose (mmol/L)**						
Baseline	7.16 (6.06, 9.30)	8.10 (6.70, 9.80)	7.45 (6.03, 8.95)	16.07 (13.92, 18.80)		<0.0001
12 months	4.38 (3.89, 5.10) *^#^	4.18 (3.35, 4.69) *^#^	4.40 (3.48, 4.80) *^#^	4.80 (3.90, 7.26) ^#^	5.79 (4.88, 6.55)	0.001
Absolute change	-2.70 (-4.92, -1.65)	-3.70 (-5.40, -2.30)	-3.00 (-3.45, -0.47)	-10.27 (-13.38, -7.83)		<0.0001
Percent change (%)	-39.5% (-53.7%, -26.1%)	-48.4% (-60.2%, -35.2%)	-38.0% (-51.3%, -8.9%)	-68.1% (-75.7%, -59.0%)		<0.0001
**Glucose_AUC_ (mmol/L•min)**						
Baseline	978 (840, 1132)	1041 (927, 1206)	1057 (932, 1198)	1764 (1556, 1998)		<0.0001
12 months	739 (644, 869) ^#^	732 (611, 837) *^#^	623 (566, 1034) ^#^	951 (817, 1286) *^#^	829 (740, 921)	<0.0001
Absolute change	-183 (-286, -79)	-275 (-398, -125)	-375 (-460, -197)	-809 (-1092, -458)		<0.0001
Percent change (%)	-18.8% (-30.4%, -8.9%)	-27.3% (-37.3%, -13.3%)	-38.4% (-46.8%, -16.0%)	-45.2% (-56.5%, -26.5%)		<0.0001
**Fasting insulin (mU/I)**						
Baseline	21.86 (15.26, 32.46)	29.76 (20.91, 41.60)	45.32 (36.51, 66.41)	21.12 (13.27, 31.86)		<0.0001
12 months	7.40 (4.63, 11.04) ^#^	8.17 (5.56, 11.59) ^#^	8.13 (4.90, 12.99) ^#^	8.47 (4.96, 13.26) ^#^	8.7 (5.65, 13.09)	0.358
Absolute change	-15.07 (-23.40, -7.73)	-23.75 (-33.79, -16.38)	-28.09 (-50.54, -18.78)	-11.30 (-19.72, -4.69)		<0.0001
Percent change (%)	-69.6% (-79.5%, -55.9%)	-77.1% (-82.1%, -63.4%)	-76.4% (-88.1%, -72.5%)	-56.8% (-76.4%, -36.2%)		<0.0001
**OGTT 2h insulin (mU/I)**						
Baseline	105.20 (63.35, 159.50)	164.55 (106.18, 247.85)	380.90 (277.70, 477.90)	54.36 (32.10, 101.70)		<0.0001
12 months	10.74 (6.41, 41.83) *^#^	11.48 (7.94, 24.87) *^#^	11.09 (7.74, 60.25) *^#^	19.21 (12.13, 36.98) *	57.78 (37.29, 91.1)	0.025
Absolute change	-63.43 (-136.33, -33.60)	-134.83 (-259.96, -95.09)	-327.93 (-433.78, -214.66)	-28.25 (-73.69, -2.74)		<0.0001
Percent change (%)	-83.8% (-94.6%, -55.4%)	-92.3% (-95.8%, -83.3%)	-96.0% (-98.1%, -87.2%)	-62.1% (-83.2%, -14.8%)		<0.0001
**Insulin_AUC_ (mU/I•min)**						
Baseline	13115 (9030, 18604)	19001 (13579, 25758)	43832 (39684, 53458)	5950 (3508, 10533)		<0.0001
12 months	8284 (5424, 16907)	12319 (6482, 20469) *^#^	14390 (6141, 33496) ^#^	5797 (3161, 10971) ^#^	6915 (5310, 10462)	0.174
Absolute change	-907 (-5250, 3949)	-7759 (-14863, 1108)	-36048 (-41321, -28410)	558 (-3465, 4818)		<0.0001
Percent change (%)	-13.1% (-50.8%, 44.2%)	-37.7% (-71.8%, 6.9%)	-72.8% (-87.9%, -52.5%)	24.6% (-40.5%, 114.2%)		<0.0001
**HBA_1_C (%)**						
Baseline	5.78 (5.40, 6.30)	5.88 (5.50, 6.30)	5.80 (5.50, 6.00)	8.10 (7.00, 9.40)		<0.0001
12 months	5.30 (5.10, 5.50) ^#^	5.20 (5.00, 5.40) ^#^	5.40 (5.10, 5.55) ^#^	5.60 (5.30, 6.00) *^#^	5.35 (5.10, 5.60)	<0.0001
Absolute change	-0.5 (-1.0, -0.2)	-0.5 (-0.9, -0.3)	-0.4 (-0.9, -0.1)	-2.1 (-3.0, -1.0)		<0.0001
Percent change (%)	-8.3% (-15.9%, -3.6%)	-9.1% (-15.3%, -5.6%)	-6.9% (-15.6%, -0.9%)	-27.2% (-34.4%, -15.1%)		<0.0001
**HOMA-IR**						
Baseline	5.09 (3.50, 7.62)	7.23 (4.71, 10.38)	9.32 (7.90, 14.53)	8.51 (5.24, 13.81)		<0.0001
12 months	1.55 (0.91, 2.25) ^#^	1.66 (1.11, 2.22) ^#^	1.60 (0.97, 2.89) ^#^	1.96 (1.15, 2.94) ^#^	1.89 (1.19, 2.88)	0.039
Absolute change	-3.53 (-6.17, -2.27)	-6.02 (-8.48, -3.79)	-6.89 (-12.99, -3.98)	-5.13 (-9.60, -2.67)		0.010
Percent change (%)	-74.4% (-82.6%, -60.4%)	-80.1% (-85.9%, -69.0%)	-80.0% (-89.9%, -71.2%)	-74.6% (-86.5%, -55.6%)		0.099
**WBISI**						
Baseline	1.94 (1.32, 2.73)	1.31 (0.94, 1.75)	0.73 (0.59, 0.87)	1.55 (1.13, 2.52)		<0.0001
12 months	5.07 (3.66, 6.80) ^#^	4.41 (3.30, 6.46) ^#^	4.83 (1.82, 7.79) ^#^	4.39 (2.94, 7.65) ^#^	4.69 (3.22, 6.75)	0.749
Absolute change	3.18 (1.13, 4.43)	3.14 (1.93, 5.06)	4.22 (1.79, 7.57)	2.40 (0.65, 6.15)		0.129
Percent change (%)	135.5% (54.9%, 232.4%)	293.2% (168.7%, 460.5%)	522.5% (233.3%, 1404.6%)	187.5% (29.7%, 423.0%)		<0.0001
**HOMA-beta**						
Baseline	272 (181, 402)	338 (240, 500)	665 (475, 1070)	86 (44, 141)		<0.0001
12 months	149 (81, 242) ^#^	178 (126, 273) ^#^	148 (109, 267) ^#^	128 (76, 212) ^#^	126 (81, 187)	0.985
Absolute change	-83 (-214, -20)	-203 (-320, -56)	-349 (-594, -149)	30 (-22, 110)		<0.0001
Percent change (%)	-38.9% (-63.3%, -10.8%)	-55.0% (-68.3%, -23.0%)	-68.1% (-76.2%, -55.1%)	36.1% (-27.6%, 171.1%)		<0.0001
**IGI**						
Baseline	25.7 (14.2, 46.8)	26.7 (15.6, 41.7)	73.8 (42.7, 102.7)	4.9 (1.8, 8.3)		<0.0001
12 months	47.3 (28.7, 75.5) *	42.6 (23.9, 70.9) *^#^	54.4 (9.7, 155.4) *	17.4 (6.2, 34.4) ^#^	17.4 (11.5, 27.2)	0.009
Absolute change	11.54 (-5.58, 42.50)	13.95 (-12.42, 40.31)	-1.93 (-47.30, 214.16)	13.91 (3.32, 34.50)		0.293
Percent change (%)	23.8% (-42.1%, 205.1%)	39.9% (-40.8%, 118.4%)	-1.8% (-84.3%, 189.0%)	285.8% (54.8%, 518.4%)		0.202
**DI (HOMA-beta/HOMA-IR)**						
Baseline	55.1 (37.4, 77.7)	49.7 (31.8, 72.1)	60.0 (41.2, 100.0)	10.4 (5.6, 16.9)		<0.0001
12 months	91.0 (66.1, 153.1) *	113.6 (79.0, 153.1) *^#^	79.8 (63.3, 113.6)	65.9 (35.7, 121.5)	68.1 (50.1, 89.4)	0.037
Absolute change	43.42 (13.05, 88.46)	53.22 (22.92, 102.52)	20.21 (-34.41, 53.64)	46.88 (24.25, 100.45)		0.230
Percent change (%)	83.6% (26.7%, 248.6%)	121.0% (38.8%, 256.8%)	27.2% (-34.0%, 123.0%)	507.1% (198.9%, 964.7%)		<0.0001
**DI (IGI · WBISI120)**						
Baseline	48.0 (24.8, 86.2)	39.2 (21.2, 67.3)	46.1 (33.1, 75.8)	5.9 (2.1, 11.6)		<0.0001
12 months	165.1 (91.7, 262.7) *^#^	165.3 (104.4, 297.1) *^#^	263.0 (66.6, 451.1) *^#^	51.2 (17.1, 117.5) ^#^	89.3 (54.4, 121.1)	<0.0001
Absolute change	101.67 (20.06, 210.00)	134.54 (54.51, 268.56)	172.62 (0.20, 451.17)	43.97 (14.49, 112.71)		0.019
Percent change (%)	181.7% (30.1%, 364.1%)	238.2% (80.4%, 639.6%)	290.0% (-0.8%, 2653.6%)	719.2% (31.2%, 1612.4%)		0.196

Values are shown as median (IQR 25-75%)), and analysis were adjusted for sex and age. MHO, metabolic healthy obesity; HMO-U, hypermetabolic obesity-hyperuricemia subtype; HMO-I, hypermetabolic obesity-hyperinsulinemia subtype; LMO, hypometabolic obesity; AUC, area under curve; HbA1c, glycosylated hemoglobin a1c; HOMA-IR, homeostatic model assessment of insulin resistance; WBISI, whole-body insulin sensitivity index; HOMA-beta, Homeostatic model assessment of beta-cell function; IGI, insulinogenic index; DI, Disposition indices; NW, normal-weight controls. *P< 0.01 vs. NW, ^#^P< 0.01 vs. baseline. ^&^ the overall ANCOVA p value.

Consistent with what we previously reported (11), patients in the HMO-I group were characterized by severe hepatic and peripheral insulin resistance as well as overcompensated insulin secretion, which resulted in a balance and made the DI and glycosylated hemoglobin a1c (HbA1c) similar to the normal-weight controls. After bariatric surgery, overcompensated insulin secretion was greatly relieved as early as at 3 months and maintained up to 12 months (median insulin_AUC_ decreased by 72.8%), together with the relief of insulin sensitivity (median WBISI increased by 5.22-folds and median HOMA-IR decreased by 80.0%) (**Table 2**). Nonetheless, the insulin secretion was still higher than that of the patients in the other three subgroups of obesity (median insulin_AUC_ 14390 vs. 5797–12319 mU/I·min in the other three subgroups) and in the NW controls (median insulin_AUC_ 6915 mU/I·min) at 12-month post-surgery (**Table 2**).

At baseline, patients in the LMO group also showed severe hepatic insulin resistance but decompensated insulin secretion, which resulted in significantly decreased DI and increased HbA1c (**Table 2**). After bariatric surgery, both insulin resistance (median HOMA-IR decreased by 74.6% and median WBISI increased by 1.87-folds) and insulin secretion (median HOMA-beta increased by 36.1%; median IGI increased by 2.85-fold) were relieved, together with a greatly relieved DI [median DI (HOMA-beta/HOMA-IR) increased by 5.07-fold, median DI (IGI · WBISI) increased by 7.19-fold] and glycemia (median glucose_AUC_ decreased by 45.2%, median HbA1c decreased by 27.2%). However, the glucose level was still higher than that of patients in the other three subgroups of obesity (median glucose_AUC_ 951 vs. 623–739 mmol/L·min and median HbA1c 5.6% vs. 5.2-5.4% in the other three subgroups) and in the NW controls (median glucose_AUC_ 828 mmol/L·min and median HbA1c 5.35%) at 12 months (**Table 2**).

For patients in MHO and HMO-U who showed relatively healthy glucometabolism pre-surgery, insulin resistance and secretion were moderately relieved, and the disposition ability of glucose was slightly improved at 12-month post-surgery.

A similar trend of post-surgery glucometabolic changes was observed when the pre-post parameter repeated measures analysis was conducted in those patients who only had the 12-month follow-up visits (**Supplementary Table S3**, **S4**; **Supplementary Figure S1**).

### The four AIM subgroups of obesity showed different remission in glucometabolic comorbidities after bariatric surgery

**Figure 2:**
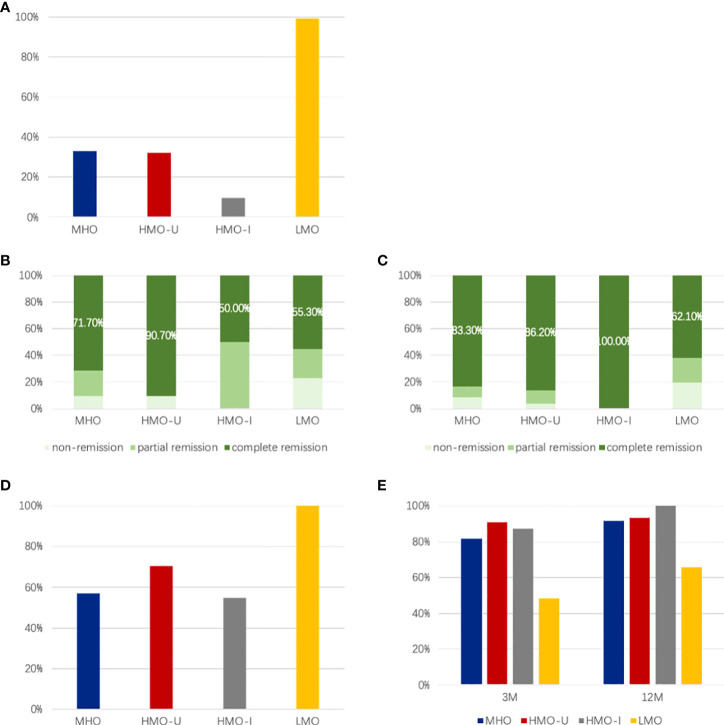
Comparison of different AIM subgroups of obesity for the rate of diabetes and hyperglycemia pre-surgery and corresponding remission rate at 3- and 12-month post-surgery. **(A)** Rate of diabetes pre-surgery; **(B, C)** Remission rate at 3-month **(B)** and 12-month **(C)** post-surgery; **(D)** Rate of hyperglycemia pre-surgery; **E,** Remission rate of hyperglycemia at 3- and 12-month post-surgery. MHO, metabolic healthy obesity; HMO-I, hypermetabolic obesity hyperinsulinemia subtype; HMO-U, hypermetabolic obesity hyperuricemia subtype; LMO, hypometabolic obesity; NW, normal-weight controls.

**Figure 2** shows the rate of diabetes pre-surgery and remission rate at 3- and 12-month post-surgery visits regarding the AIM subtypes of obesity. At baseline (**Figure 2A**), 49.7% of patients with obesity had diabetes, among which the LMO patients showed the highest rate (99.2% vs. 9.5–34.0% in the other three subgroups), which was consistent with what we reported previously (11). At 12-month post-surgery (**Figure 2C**), 83.3%, 86.2%, 100%, and 62.1% of patients with diabetes pre-surgery achieved complete remission in MHO, HMO-U, HMO-I, LMO, respectively, according to the ADA criteria (18), with patients in LMO having the lowest remission rate. At baseline (**Figure 2D**), 72.6% of patients with obesity had hyperglycemia, and patients in LMO showed the highest rate (100% vs. 54.8–70.4% in the other three subgroups). At 12-month post-surgery (**Figure 2E**), 91.5%, 93.5%, 100%, and 65.7% of patients with hyperglycemia pre-surgery achieved remission in MHO, HMO-U, HMO-I, and LMO, respectively, with patients in LMO having the lowest remission rate. Thirteen patients (n=1, 5, 0, 7 for MHO, HMO-U, HMO-I, and LMO, respectively) used anti-diabetes medication because of inadequate glucose control or insulin resistance.

**Figure 3:**
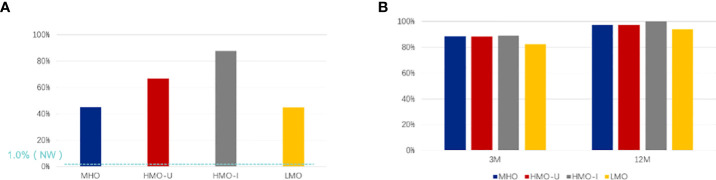
Comparison of different AIM subgroups of obesity for the rate of hyperinsulinemia pre-surgery **(A)** and remission rate post-surgery **(B)**. **(A)** The rate of hyperinsulinemia pre-surgery; **(B)** Remission rate of hyperinsulinemia at 3-month (3M) and 12-month (12M) post-surgery. MHO, metabolic healthy obesity; HMO-I, hypermetabolic obesity hyperinsulinemia subtype; HMO-U, hypermetabolic obesity hyperuricemia subtype; LMO, hypometabolic obesity; NW, normal weight control.

At baseline (**Figure 3A**), 54.4% of patients with obesity had hyperinsulinemia, in which patients in HMO-I showed the highest rate (87.8% vs. 45.1–66.7% in the other three subgroups), consistent with what we reported previously (11). At 12-month after surgery, the rate of hyperinsulinemia decreased to 3.1% for the full cohort, which was comparable to the NW controls (1.0%, P = 0.413). Among the patients with hyperinsulinemia pre-surgery, 97.1%, 97.2%, 100%, and 94.1% achieved remission in MHO, HMO-U, HMO-I, and LMO, respectively. The remission rate of hyperinsulinemia was similar among the four AIM subgroups (**Figure 3B**).

**Figure 4:**
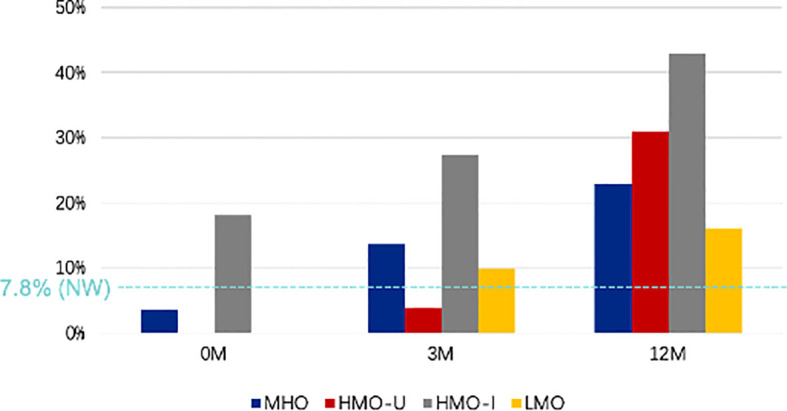
Comparison of different AIM subgroups of obesity for the rate of hypoglycemia during OGTT at baseline and at 3- and 12-month post-surgery. MHO, metabolic healthy obesity; HMO-I, hypermetabolic obesity hyperinsulinemia subtype; HMO-U, hypermetabolic obesity hyperuricemia subtype; LMO, hypometabolic obesity; NW, normal weight control; 0M, pre-surgery; 3M, 3-month post-surgery; 12M, 12-month post-surgery.

At baseline (**Figure 4**), 2.5% of patients with obesity had hypoglycemia during OGTT; among them, patients in HMO-I showed the highest rate (18.2% vs. 0–3.6% in the other three subgroups), consistent with the highest rate of hyperinsulinemia observed in this group. The incidence of hypoglycemia during OGTT increased to 24.8% in all the obese patients at 12-month post-surgery (**Figure 4**), which was significantly higher than that in the NW controls (7.8%, P = 0.001). Patients in the HMO-I group still showed the highest rate of hypoglycemia during OGTT (42.9% vs. 16.2–31.0% in the other three subgroups), even with a great improvement of hyperinsulinemia.

Similar observations of diabetes and hyperinsulinemia remission, and rate of hypoglycemia were reported when patients who underwent RYGB and LSG were analysed separately (**Supplementary Figure S2**, **S3**).

## Discussion

According to the current study, the four AIM subtypes of obesity benefited differently on glucometabolism from bariatric surgery. In particular, patients in LMO showed the highest incidence of diabetes before surgery and the least diabetes remission rate at 12-month post-surgery. Nonetheless, a substantial post-surgery reduction in blood glucose was observed for these patients. Patients in HMO-I had severe hyperinsulinemia before surgery, which resolved 12 months later. However, these patients were still most prone to the postoperative complications of hypoglycemia. For patients in MHO and HMO-U who showed a relative healthy glucometabolism before surgery, glucometabolic comorbidities improved moderately after surgery.

In current clinic, there are different diagnostic categories for obesity [e.g. BMI categories, healthy/unhealthy obesity (20), ABCD categories (21), obesity phenotype classification (22)] and various criteria for evaluating bariatric surgery (6). There are also several established scoring systems [e.g. DiaRem score (23) and ABCD score (24)] showing that a set of factors (e.g., age, disease duration, pancreatic beta-cell function) are predictive of the effects of bariatric surgery (23–26). It should be pointed out that, the AIM subtyping system is not meant to replace the existing mechanisms in guiding obesity therapy. Instead, our goal with the AIM subtypes is to explore machine learning-based refined clustering of obesity patients and then to correlate the four subtypes with the effects of bariatric surgery. Continuous to the encouraging findings in our previous work (11), this study provided additional clinical indications on the AIM-based patient classification with regards to glucometabolic outcomes due to the bariatric surgery. Our findings here thus have the potential to better inform clinicians in rendering clinical decisions of bariatric surgery and in counseling patients regarding potential postoperative outcomes.

Bariatric surgery has become the most recommended treatment for morbid obesity with T2DM over lifestyle intervention and medical therapy in recent years (27). Plenty studies have demonstrated that bariatric surgery could alleviate diabetes (remission rate 30–95% in 1 to 5- year follow-up) (3–5, 7, 8), decrease the risk of microvascular and macrovascular complications (5), and increase life expectancy (by 9.3-year) (28). In the current study, the overall complete remission rate of diabetes after surgery was 85.5%, among which LMO showed the lowest remission rate despite the highest incidence before surgery. We suspect this observation may be related to the oldest age and the worst pancreatic beta-cell function, the previously-shown predictive factors of poor remission rate (26), at both baseline and 12-month post-surgery for patients in the LMO group. This finding implies an important research question to further investigate whether patients in LMO may be benefiting from a bariatric surgery. Note that although 37.9% of patients did not achieve complete diabetes remission, a remarkable reduction in blood glucose (absolute value of HbA1c reduced 2.1%) were still observed for LMO. It is well known that the blood glucose level is positively related to the chronic complications of diabetes, as each 1% decline in HbA1c is associated with a 21% decrease in diabetes end-point events (29). Thus, for the purpose of preventing chronic complications of diabetes, patients in LMO may be more proactively considered for a bariatric procedure. This will require discretion from clinicians in counseling those patients about potential postoperative outcomes.

Bariatric surgery is also an effective therapeutic method for insulin resistance and hyperinsulinemia, which have a reciprocal relationship and act as the underlying mechanism of various metabolic disorders (30–32). Meta-analysis had reported that bariatric surgery reduces insulin resistance within 2 weeks and lasts 12 months after surgery (HOMA-IR reduced to 33.48 ± 5.78% and 44.91 ± 7.98% of the baseline, respectively) (9). The mechanism of improved insulin resistance after bariatric surgery was not fully understood yet. While weight loss and caloric restriction play important roles, it remains a complex process to interpret and needs further investigation. Other potential reasons may include increased gastrointestinal hormone release, reduced production of adipose inflammatory factors, changes in circulating bile acid or branched amino acid concentrations, and changes in the gut microbiome (33, 34). In the present study, the overall average remission rate of hyperinsulinemia was as high as 96.9%. Although HMO-I had the highest preoperative hyperinsulinemia incidence, it was resolved at 12-month post-surgery, as well as a remarkable relief of insulin resistance. Thus, bariatric surgery may be highly recommended for patients in HMO-I. Although the rate of hyperinsulinemia was low in patients of LMO, their insulin resistance was still severe (HOMA-IR 8.51) but greatly remitted at 12-month after surgery (HOMA-IR decreased to 1.96, similar to that of NW). Post-surgery improvement in insulin resistance may be another potential mechanism of diabetes remission in LMO.

Hypoglycemia, especially postprandial hypoglycemia, is one of the most common complications of bariatric surgery. Reportedly, 1/3 to 2/3 of patients had experienced postprandial hypoglycemia after bariatric surgery (2, 35). Possible reasons include reduced food intake, improper secretion of insulin and gastrointestinal hormones, and resetting of glucose-regulating homeostasis after the surgery (36–38). In the current study, the overall average incidence of hypoglycemia during OGTT 12-month after surgery was 24%, with HMO-I being the highest. For HMO-I, although fasting hyperinsulinemia was relieved after surgery, glucose stimulated insulin release was sharp, especially within 60 minutes after glucose stimulation, which is consistent with previous studies (39). In addition, we observed a more rapid relief of insulin resistance (WBISI increased 5.22-fold) than glucose induced insulin secretion (IGI only reduced by 1.8%). The unbalanced relief rate of insulin resistance and secretion resulted in a significantly higher DI in HMO-I at 12-month post-surgery than that in NW (DI[IGI·WBISI] 263.0 vs. 89.3). Thus, we speculated that, relative to the sharp weight loss, the recovery of obesity-induced compensatory pancreatic β cell proliferation (40) may have been delayed, leading to a severe improper postprandial secretion of insulin after surgery for HMO-I. This may be a potential explanation for the high incidence of hypoglycemia during OGTT in HMO-I. The high incidence of postprandial hypoglycemia in HMO-I may require attention of clinicians in post-surgery care, where nutrition consultation may also be advised for those patients.

Of note, a recent study reported a similar observation on the bariatric surgery outcomes within different diabetes/obesity subtypes. In that study (40), patients in severe insulin resistance subgroup were associated with more improvement of insulin resistance, hyper fasting insulin, and more frequent diabetes remission; and patients in severe insulin deficient subgroup were associated with more reduction of HbA1c and fasting glucose yet lower diabetes remission after surgery (41). With different methods and populations for the diabetes/obesity subtyping, the similar findings of our work contribute to further insights on evaluating the outcomes of bariatric surgery on subtyped patients.

Our study has some limitations. Since this was a multicenter retrospective study, potential bias may have been introduced in certain aspects. First, there are two types of surgery (i.e., LSG and RYBG) conducted on our study cohort. We observed similar outcomes though when separately analyzing patients underwent RYGB and LSG. A recent meta-analysis of 32 studies including 2475 patients also reported a similar remission rate of diabetes and insulin resistance for LSG and RYBG (42). Second, the durability profile and diabetes relapse were uncertain due to the relatively short follow-up time periods. Third, considering OGTT may cause the post prandial hypoglycemia episodes as glucose strongly stimulates the release of insulin, it is possible that the incidence of hypoglycemia was overestimated especially at post surgery visits. In addition, we acknowledge that our study has a low follow-up rate, which had to do with multiple factors including patient compliance, health system, and influence of COVID-19. The smaller size of the HMO-I group may not represent the normal diabete remission rate. Future prospective studies with larger cohorts, longer follow-ups, and better protocol design are needed to further evaluate effects of the bariatric outcomes of the AIM subtypes of obesity.

In summary, this study examines effects of the four AIM subtypes and yields the following indications to the HMO-I and LMO subgroups. HMO-I was characterized by preoperative hyperinsulinemia which remitted after surgery. Bariatric surgery may be recommended for these patients, yet post-surgery hypoglycemia should be concerned. LMO had the highest rate of diabetes before surgery. Although the remission rate of diabetes after surgery was lower than the other subgroups, their blood glucose could be reduced remarkably. In this sense, LMO may be more motivated for a bariatric procedure, with appropriate counseling to patients about potential postoperative outcomes.

## Conclusion

We performed a multicenter study to evaluate effects of glucometabolic response to bariatric surgery for patients categorized by the four AIM subtypes. In terms of glucometabolism, the four AIM subtypes of patients benefited differently from a bariatric surgery, which significantly relieved hyperglycemia and hyperinsulinemia for the LMO and HMO-I patients, respectively. The AIM-based subtypes may help better inform clinical decisions on bariatric surgery and patient counseling pertaining to post-surgery outcomes. Future studies from other perspectives of metabolic outcomes may further evaluate the benefits of bariatric surgery with respect to the subtypes of obese patients.

## Data availability statement

The original contributions presented in the study are included in the article/**Supplementary Material**. Further inquiries can be directed to the corresponding authors.

## Ethics statement

The studies involving human participants were reviewed and approved by The Ethical Committee of Shanghai Tenth People’s Hospital. The patients/participants provided their written informed consent to participate in this study.

## Author contributions

YL, CS, and ZL analyzed and interpreted the data and wrote the manuscript. YL, LS, JL, and HY collected the data. WF, FS, JZ, and YC provided multi-center data. LD and XJ evaluated and implemented bariatric surgery. SQ and ZL designed the study and revised the manuscript. SW provided input in machine learning and edited the manuscript. All authors approved the final version of the manuscript. SQ and ZL are the guarantors of this work and, as such, have full access to all the data in the study and take responsibility for the integrity of the data and the accuracy of the data analysis. All authors contributed to the article and approved the submitted version.

## Funding

This study was supported by the Clinical Research Plan of SHDC (No. SHDC2020CR1017B), National Natural Science Foundation of China (82170861, 81970677), Shanghai Medicine and Health Development Foundation (DMRFP_I_07), Shanghai Commission of Science and Technology (19DZ1910200). Funding sources had no involvement in study design, the collection, analysis, or interpretation of data, the report’s writing, or the decision to submit the paper for publication.

## Acknowledgments

We thank all patients included in this study. In addition, we thank the involved healthcare providers and students for their support to this study. A half-page abstract of this study was accepted to present as a poster at the 82th Scientific Sections of American Diabetes Association (ADA) in June 2022.

## Conflict of interest

The authors declare that the research was conducted in the absence of any commercial or financial relationships that could be construed as a potential conflict of interest.

## Publisher’s note

All claims expressed in this article are solely those of the authors and do not necessarily represent those of their affiliated organizations, or those of the publisher, the editors and the reviewers. Any product that may be evaluated in this article, or claim that may be made by its manufacturer, is not guaranteed or endorsed by the publisher.
