# Supplementary material for: A multi-center study on glucometabolic response to bariatric surgery for different subtypes of obesity
Source: Front Endocrinol (Lausanne). 2022 Nov 3;13:989202. doi: 10.3389/fendo.2022.989202 (PMC9669340; doi:10.3389/fendo.2022.989202)
Supplement: Supplementary file 1 [file DataSheet_1.pdf]

Supplementary Material

A multi-center study on glucometabolic response to bariatric surgery for different subtypes of obesity

Yao Liu[1†], Chunjun Sheng[1†], Wenhuan Feng[2], Fang Sun[3], Jingjing Zhang[4], Ying Chen[5], Lili Su[1], Jia Liu[1], Lei Du[1], Xuyang Jia[1], Hui You[1], Xiu Huang[1], Shandong Wu[6], Ziwei Lin[1*], and Shen Qu[1*]

[1]Department of Endocrinology and Metabolism, Shanghai Tenth People's Hospital, Tongji University School of Medicine, Shanghai 200072, China

[2]Department of Endocrinology, Drum Tower Hospital Affiliated to Nanjing University Medical School, Nanjing, 210008, China

[3]Department of Hypertension and Endocrinology, Daping Hospital, Army Medical University, Chongqing Institute of Hypertension, Chongqing 400042, China.

[4]National Clinical Research Center for Metabolic Diseases, Metabolic Syndrome Research Center, Key Laboratory of Diabetes Immunology, Ministry of Education, and Department of Metabolism and Endocrinology, The Second Xiangya Hospital of Central South University, Changsha 410011, Hunan, China

[5]Ministry of Education Key Laboratory of Metabolism and Molecular Medicine, Department of Endocrinology and Metabolism, Zhongshan Hospital, Fudan University, Shanghai 200032, China.

[6]Department of Radiology, Department of Biomedical Informatics, Department of Bioengineering, Intelligent Systems Program, University of Pittsburgh, Pittsburgh, PA, United States

†These authors contributed equally to this work and share first authorship.
*Correspondence: Ziwei Lin (Email: lin_ziwei225@163.com) and Shen Qu (Email: qushencn@hotmail.com)

Supplementary Methods

AIM grouping of patients with obesity

The modeling method for AIM subgrouping has been described in our previous study[1]. Briefly, obesity patients were clustered into four subgroups (i.e., MHO, HMO-U, HMO-I, and LMO) using the k-means algorithm. Men and women were separately modeled with two sub-models. The key variables used for the k-means clustering were glucoseAUC, insulinAUC, and uric acid (cluster centers were shown in Table S1).

Missing value imputation

The linear regression model was trained and tested in 70% and 30%, respectively, of the data from Cohort-1 using the stepwise method. The F test was performed with $P < 0.05$ for inclusion and $P > 0.1$ for exclusion with the outlier tolerance of 0.0001. InsulinAUC showed an adjusted R of 0.902 and 0.903 in the training and testing datasets, respectively (Table S2).

References

1. Lin Z, Feng W, Liu Y, et al. Machine Learning to Identify Metabolic Subtypes of Obesity: A Multi-Center Study. *Front Endocrinol (Lausanne).* 2021;12:713592.

Supplementary Tables

Table S1. Cluster centers for k-means model.

	MHO	HMO-U	HMO-I	LMO
**Sub-model for Men**				
Glucose $_{AUC}$, mmol/L·min	993	982	1008	1845
Insulin $_{AUC}$, mU/L·min	17089	19951	50543	9477
Uric acid, µmol/L	405	585	489	404
**Sub-model for Women**				
Glucose $_{AUC}$, mmol/L·min	928	1100	1094	1829
Insulin $_{AUC}$, mU/L·min	12894	21895	54645	9320
Uric acid, µmol/L	331	454	392	356

AUC: area under the curve during oral glucose tolerance test; HMO-U: hypermetabolic obesity-hyperuricemia subtype; HMO-I: hypermetabolic obesity-hyperinsulinemia subtype; LMO: hypometabolic obesity; MHO: metabolic healthy obesity.

Table S2. Regression models for prediction of AUC of insulin during OGTT.

Predictors	*β*	Standardized *β*	*t* test	*P*	Adjusted R in training dataset	Adjusted R in testing dataset
**Constant**	-13032.851		-17.477	<0.001		
**C-peptide $_{60min}$**	1309.195	0.601	22.473	<0.001		
**C-peptide $_{120min}$**	439.883	0.177	9.644	<0.001		
**C-peptide $_{30min}$**	656.368	0.273	12.859	<0.001	0.902	0.903
**Glucose $_{120min}$**	710.316	0.213	7.395	<0.001		
**Glucose $_{60min}$**	-562.742	-0.157	-6.072	<0.001		
**C-peptide $_{0min}$**	467.843	0.057	3.592	<0.001		

Models were trained with Cohort-1 patients, among which 70% of the samples were used as training datasets and 30% as testing datasets. AUC: area under the curve; OGTT: oral glucose tolerance test.

Table S3. The anthropometry characteristics of the four AIM subgroups of obesity patients at baseline and 12-month post-surgery.

	MHO	HMO-U	HMO-I	LMO	p value[&]
**N**	118	111	14	116	
**Man/Woman (woman %)**	50/68(57.6%)	49/62(55.9%)	8/6(42.9%)	50/66(56.9%)	0.055
**Age (years)**	32(27,42)	28(23,35)	32(25,35)	40(31,48)	<0.0001
**Obesity duration (years)**	10.0(5.5,20.0)	10.5(5.8,20.0)	10.0(5.0,20.0)	13.0(9.0,20.0)	0.129
**Type of surgery**	28.0%	15.3%	14.3%	44.8%	<0.0001
**Rate of diabetes (%)**	37.4%	29.1%	7.1%	100%	<0.0001
**Diabetes duration (years)**	1.0(0,4.0)	0(0,1.8)	0(0,0)	1.0(0,5.5)	0.193
**Weight (kg)**					
Baseline	104.3 (91.7, 128.2)	116.9 (98.3, 133.5)	119.0 (100.5, 132.3)	98.3 (82.3, 113.9)	<0.0001
12 months	74.1 (64.1, 87.3) [#]	78.4 (66.9, 89.1) [#]	80.2 (71.1, 95.9)	71.7 (62.1, 83.2) [#]	0.354
Absolute change	-31.6 (-41.8, -23.7)	-37.8 (-47.2, -31.3)	-37.4 (-53.8, -31.9)	-24.6 (-31.8, -17.1)	<0.0001
Percent change (%)	-29.4% (-35.7%, -25.0%)	-33.5% (-38.3%, -26.9%)	-34.0% (-37.6%, -25.0%)	-24.4% (-30.2%, -20.3%)	<0.0001
**BMI (kg/m^2)**					
Baseline	37.8 (34.4, 42.2)	40.1 (35.8, 45.4)	39.1 (35.8, 42.3)	35.5 (30.6, 40.6)	0.001
12 months	26.9 (23.5, 30.1) [#]	27.0 (24.5, 30.3) [#]	27.1 (24.7, 29.1)	26.7 (23.3, 29.4) [#]	0.781
Absolute change	-11.1 (-13.9, -9.2)	-13.3 (-16.3, -10.8)	-12.5 (-16.2, -9.2)	-8.9 (-11.3, -6.4)	<0.0001
Percent change (%)	-29.4% (-35.7%, -25.0%)	-33.5% (-38.3%, -26.9%)	-34.0% (-37.6%, -25.0%)	-24.4% (-30.2%, -20.3%)	<0.0001
**Excess weight (kg)**					
Baseline	39.8 (28.7, 56.0)	45.8 (33.0, 62.0)	44.3 (33.4, 57.3)	31.9 (18.4, 48.1)	<0.0001
12 months	8.1 (-1.3, 17.4) [#]	8.2 (1.4, 17.0) [#]	10.3 (1.9, 16.9)	7.2 (-2.1, 15.9) [#]	0.696
Absolute change	-31.6 (-41.8, -23.7)	-37.8 (-47.2, -31.3)	-37.4 (-53.8, -31.9)	-24.6 (-31.8, -17.1)	<0.0001
Percent change (%)	-81.1% (-105.6%, -66.2%)	-83.1% (-96.6%, -68.7%)	-79.3% (-95.8%, -65.9%)	-77.7% (-111.8%, -62.0%)	0.756
**WC (cm)**					
Baseline	117 (105, 129)	121 (112, 130)	125 (109, 139)	113 (103, 125)	0.009
12 months	89 (82, 101)	92 (85, 98)	90 (84, 96)	92 (83, 98) [#]	0.885
Absolute change	-27 (-35, -19)	-31 (-35, -23)	-30 (-46, -26)	-21 (-29, -14)	<0.0001
Percent change (%)	-22.8% (-28.6%, -16.6%)	-23.9% (-29.9%, -19.5%)	-26.7% (-30.9%, -22.0%)	-18.2% (-23.7%, -14.8%)	<0.0001
**HC (cm)**					
Baseline	120 (113, 129)	122 (115, 132)	122 (112, 131)	114 (105, 124)	<0.0001
12 months	101 (95, 106) [#]	100 (94, 107) [#]	103 (98, 107)	98 (91, 105) [#]	0.486
Absolute change	-20 (-25, -13)	-23 (-28, -16)	-19 (-33, -11)	-13 (-19, -8)	<0.0001
Percent change (%)	-16.0% (-19.7%, -11.6%)	-18.3% (-21.6%, -12.7%)	-15.3% (-22.7%, -9.5%)	-11.6% (-15.0%, -7.7%)	<0.0001

	MHO	HMO-U	HMO-I	LMO	p value[&]
**WHR (ratio)**					
Baseline	0.98 (0.91, 1.03)	0.98 (0.94, 1.03)	1.02 (0.98, 1.06)	0.99 (0.95, 1.04)	0.129
12 months	0.91 (0.85, 0.95)	0.90 (0.87, 0.95)	0.88 (0.83, 0.93)	0.92 (0.88, 0.97)	0.065
Absolute change	-0.08 (-0.12, -0.03)	-0.08 (-0.13, -0.04)	-0.13 (-0.18, -0.10)	-0.07 (-0.10, -0.04)	0.065
Percent change (%)	-8.2% (-11.8%, -3.7%)	-8.3% (-12.2%, -3.9%)	-12.8% (-16.8%, -10.8%)	-6.7% (-10.4%, -4.1%)	0.062

Analysis are in only those patients having the 12-month follow-up visit. Values are shown as ratio or median (IQR 25-75%), and analysis were adjusted for sex and age (except analysis for sex and age). MHO: metabolic healthy obesity; HMO-U: hypermetabolic obesity-hyperuricemia subtype; HMO-I: hypermetabolic obesity-hyperinsulinemia subtype; LMO: hypometabolic obesity; BMI, body mass index; WC, waist circumference; HC, hip circumference; WHR, Waist-to-Hip Ratio. [#]P< 0.01 vs. baseline; [&] the overall ANCOVA p values.

Table S4. The glucometabolism of the four AIM subgroups of obesity and normal weight controls at baseline and 12-month post-surgery.

	MHO	HMO-U	HMO-I	LMO	NW	P value[&]
**Fasting glucose (mmol/L)**						
Baseline	5.20 (4.77, 5.93)	5.20 (4.78, 5.92)	5.20 (4.65, 5.53)	8.28 (6.86, 10.55)		<0.0001
12 months	4.47 (4.10, 4.76) *[#]	4.40 (4.15, 4.70) *[#]	4.60 (4.40, 4.91)	4.87 (4.38, 5.70) [#]	4.86 (4.60, 5.22)	<0.0001
Absolute change	-0.63 (-0.27, -1.35)	-0.80 (-0.30, -1.50)	-0.60 (0.15, -1.12)	-3.46 (-1.70, -5.56)		<0.0001
Percent change (%)	-13.3% (-23.5%, -5.9%)	-15.3% (-25.9%, -6.4%)	-12.0% (-19.4%, 3.4%)	-40.9% (-53.6%, -27.4%)		<0.0001
**OGTT 2h glucose (mmol/L)**						
Baseline	7.20 (6.20, 9.10)	8.00 (6.41, 9.45)	6.95 (5.31, 7.75)	15.90 (14.10, 18.75)		<0.0001
12 months	4.38 (3.89, 5.10) *[#]	4.18 (3.35, 4.69) *[#]	4.40 (3.48, 4.80) *	4.80 (3.90, 7.26) [#]	5.79 (4.88, 6.55)	0.001
Absolute change	-2.70 (-4.92, -1.65)	-3.70 (-5.40, -2.30)	-3.00 (-3.45, -0.47)	-10.27 (-13.38, -7.83)		<0.0001
Percent change (%)	-39.5% (-53.7%, -26.1%)	-48.4% (-60.2%, -35.2%)	-38.0% (-51.3%, -8.9%)	-68.1% (-75.7%, -59.0%)		<0.0001
**$Glucose_{AUC}$ (mmol/L•min)**						
Baseline	980 (825, 1131)	1028 (921, 1172)	1080 (947, 1209)	1711 (1511,2009)		<0.0001
12 months	739 (644, 869)	732 (611, 837) *	623 (566, 1034) [#]	951 (817, 1286) *[#]	829 (740, 921)	<0.0001
Absolute change	-183 (-286, -79)	-275 (-398, -125)	-375 (-460, -197)	-809 (-1092, -458)		<0.0001
Percent change (%)	-18.8% (-30.4%, -8.9%)	-27.3% (-37.3%, -13.3%)	-38.4% (-46.8%, -16.0%)	-45.2% (-56.5%, -26.5%)		<0.0001
**Fasting insulin (mU/I)**						
Baseline	21.78 (14.29, 32.31)	32.15 (24.05, 43.46)	45.32 (26.00, 68.32)	18.97 (12.77, 27.07)		<0.0001
12 months	7.40 (4.63, 11.04) [#]	8.17 (5.56, 11.59) [#]	8.13 (4.90, 12.99)	8.47 (4.96, 13.26)	8.7 (5.65, 13.09)	0.358
Absolute change	-15.07 (-23.40, -7.73)	-23.75 (-33.79, -16.38)	-28.09 (-50.54, -18.78)	-11.30 (-19.72, -4.69)		<0.0001
Percent change (%)	-69.6% (-79.5%, -55.9%)	-77.1% (-82.1%, -63.4%)	-76.4% (-88.1%, -72.5%)	-56.8% (-76.4%, -36.2%)		<0.0001

	MHO	HMO-U	HMO-I	LMO	NW	P value&
**OGTT 2h insulin (mU/I)**						
Baseline	94.83 (60.17, 152.50)	162.15 (119.48, 260.40)	359.80 (271.25, 467.50)	48.44 (31.23, 94.17)		<0.0001
12 months	10.74 (6.41, 41.83) *#	11.48 (7.94, 24.87) *	11.09 (7.74, 60.25) *#	19.21 (12.13, 36.98) *	57.78 (37.29, 91.1)	0.025
Absolute change	-63.43 (-136.33, -33.60)	-134.83 (-259.96, -95.09)	-327.93 (-433.78,	-28.25 (-73.69, -2.74)		<0.0001
Percent change (%)	-83.8% (-94.6%, -55.4%)	-92.3% (-95.8%, -83.3%)	-96.0% (-98.1%, -87.2%)	-62.1% (-83.2%, -14.8%)		<0.0001
**Insulin$_{AUC}$ (mU/I •min)**						
Baseline	12335 (8658, 17630)	20134 (14392, 27719)	45680 (42455, 52353)	5454 (3243, 10121)		<0.0001
12 months	8284 (5424, 16907) #	12319 (6482, 20469) *	14390 (6141, 33496) #	5797 (3161, 10971) #	6915 (5310, 10462)	0.174
Absolute change	-907 (-5250, 3949)	-7759 (-14863, 1108)	-36048 (-41321, -28410)	558 (-3465, 4818)		<0.0001
Percent change (%)	-13.1% (-50.8%, 44.2%)	-37.7% (-71.8%, 6.9%)	-72.8% (-87.9%, -52.5%)	24.6% (-40.5%, 114.2%)		<0.0001
**HBA$_1$C (%)**						
Baseline	5.7 (5.5, 6.3)	5.8 (5.5, 6.2)	5.8 (5.5, 6.0)	7.9 (6.8, 9.3)		<0.0001
12 months	5.30 (5.10, 5.50) #	5.20 (5.00, 5.40) #	5.40 (5.10, 5.55)	5.60 (5.30, 6.00) *	5.35 (5.10, 5.60)	<0.0001
Absolute change	-0.5 (-1.0, -0.2)	-0.5 (-0.9, -0.3)	-0.4 (-0.9, -0.1)	-2.1 (-3.0, -1.0)		<0.0001
Percent change (%)	-8.3% (-15.9%, -3.6%)	-9.1% (-15.3%, -5.6%)	-6.9% (-15.6%, -0.9%)	-27.2% (-34.4%, -15.1%)		<0.0001
**HOMA-IR**						
Baseline	5.11 (3.47, 7.98)	7.53 (5.88, 10.89)	9.17 (5.55, 16.18)	7.46 (4.74, 11.48)		<0.0001
12 months	1.55 (0.91, 2.25)	1.66 (1.11, 2.22)	1.60 (0.97, 2.89)	1.96 (1.15, 2.94)	1.89 (1.19, 2.88)	0.039
Absolute change	-3.53 (-6.17, -2.27)	-6.02 (-8.48, -3.79)	-6.89 (-12.99, -3.98)	-5.13 (-9.60, -2.67)		0.010
Percent change (%)	-74.4% (-82.6%, -60.4%)	-80.1% (-85.9%, -69.0%)	-80.0% (-89.9%, -71.2%)	-74.6% (-86.5%, -55.6%)		0.099
**WBISI**						
Baseline	2.03 (1.39, 2.94)	1.20 (0.92, 1.52)	0.75 (0.62, 0.88)	1.73 (1.19, 2.78)		<0.0001
12 months	5.07 (3.66, 6.80)	4.41 (3.30, 6.46)	4.83 (1.82, 7.79) #	4.39 (2.94, 7.65)	4.69 (3.22, 6.75)	0.749
Absolute change	3.18 (1.13, 4.43)	3.14 (1.93, 5.06)	4.22 (1.79, 7.57)	2.40 (0.65, 6.15)		0.129
Percent change (%)	135.5% (54.9%, 232.4%)	293.2% (168.7%, 460.5%)	522.5% (233.3%, 1404.6%)	187.5% (29.7%, 423.0%)		<0.0001
**HOMA-beta**						
Baseline	249 (161, 360)	361 (240, 524)	567 (325, 892)	85 (44, 136)		<0.0001
12 months	149 (81, 242)	178 (126, 273) #	148 (109, 267) #	128 (76, 212) #	126 (81, 187)	0.985
Absolute change	-83 (-214, -20)	-203 (-320, -56)	-349 (-594, -149)	30 (-22, 110)		<0.0001
Percent change (%)	-38.9% (-63.3%, -10.8%)	-55.0% (-68.3%, -23.0%)	-68.1% (-76.2%, -55.1%)	36.1% (-27.6%, 171.1%)		<0.0001
**IGI**						
Baseline	25.7 (16.7, 51.5)	30.0 (20.9, 47.2)	60.8 (40.4, 93.5)	5.0 (2.2, 7.7)		<0.0001
12 months	47.3 (28.7, 75.5) *	42.6 (23.9, 70.9) *	54.4 (9.7, 155.4) *	17.4 (6.2, 34.4) #	17.4 (11.5, 27.2)	0.009
Absolute change	11.54 (-5.58, 42.50)	13.95 (-12.42, 40.31)	-1.93 (-47.30, 214.16)	13.91 (3.32, 34.50)		0.293

	MHO	HMO-U	HMO-I	LMO	NW	P value[&]
Percent change (%)	23.8% (-42.1%, 205.1%)	39.9% (-40.8%, 118.4%)	-1.8% (-84.3%, 189.0%)	285.8% (54.8%, 518.4%)		0.202
**DI (HOMA-beta / HOMA-IR)**						
Baseline	50.9 (31.2, 65.9)	48.5 (31.8, 72.1)	55.1 (39.6, 90.5)	10.9 (6.6, 18.6)		<0.0001
12 months	91.0 (66.1, 153.1) *#	113.6 (79.0, 153.1) *#	79.8 (63.3, 113.6)	65.9 (35.7, 121.5) #	68.1 (50.1, 89.4)	0.037
Absolute change	43.42 (13.05, 88.46)	53.22 (22.92, 102.52)	20.21 (-34.41, 53.64)	46.88 (24.25, 100.45)		0.230
Percent change (%)	83.6% (26.7%, 248.6%)	121.0% (38.8%, 256.8%)	27.2% (-34.0%, 123.0%)	507.1% (198.9%, 964.7%)		<0.0001
**DI (IGI · WBISI120)**						
Baseline	53.3 (30.6, 93.7)	39.8 (25.4, 65.1)	44.5 (33.0, 83.7)	6.8 (3.4, 12.2)		<0.0001
12 months	165.1 (91.7, 262.7) *#	165.3 (104.4, 297.1) *#	263.0 (66.6, 451.1) *#	51.2 (17.1, 117.5) #	89.3 (54.4, 121.1)	<0.0001
Absolute change	101.67 (20.06, 210.00)	134.54 (54.51, 268.56)	172.62 (0.20, 451.17)	43.97 (14.49, 112.71)		0.019
Percent change (%)	181.7% (30.1%, 364.1%)	238.2% (80.4%, 639.6%)	290.0% (-0.8%, 2653.6%)	719.2% (31.2%, 1612.4%)		0.196

Analysis are in only those patients having the 12-month follow-up visit. Values are shown as median (IQR 25-75%)), and analysis were adjusted for sex and age. MHO: metabolic healthy obesity; HMO-U: hypermetabolic obesity-hyperuricemia subtype; HMO-I: hypermetabolic obesity-hyperinsulinemia subtype; LMO: hypometabolic obesity; AUC, area under curve; HbA1c, glycosylated hemoglobin a1c; HOMA-IR, homeostatic model assessment of insulin resistance; WBISI, whole-body insulin sensitivity index; HOMA-beta, Homeostatic model assessment of beta-cell function; IGI, insulinogenic index; DI, Disposition indices; NW, normal-weight controls. *P< 0.01 vs. NW, #P< 0.01 vs. baseline. & the overall ANCOVA p values.

Supplementary Figures

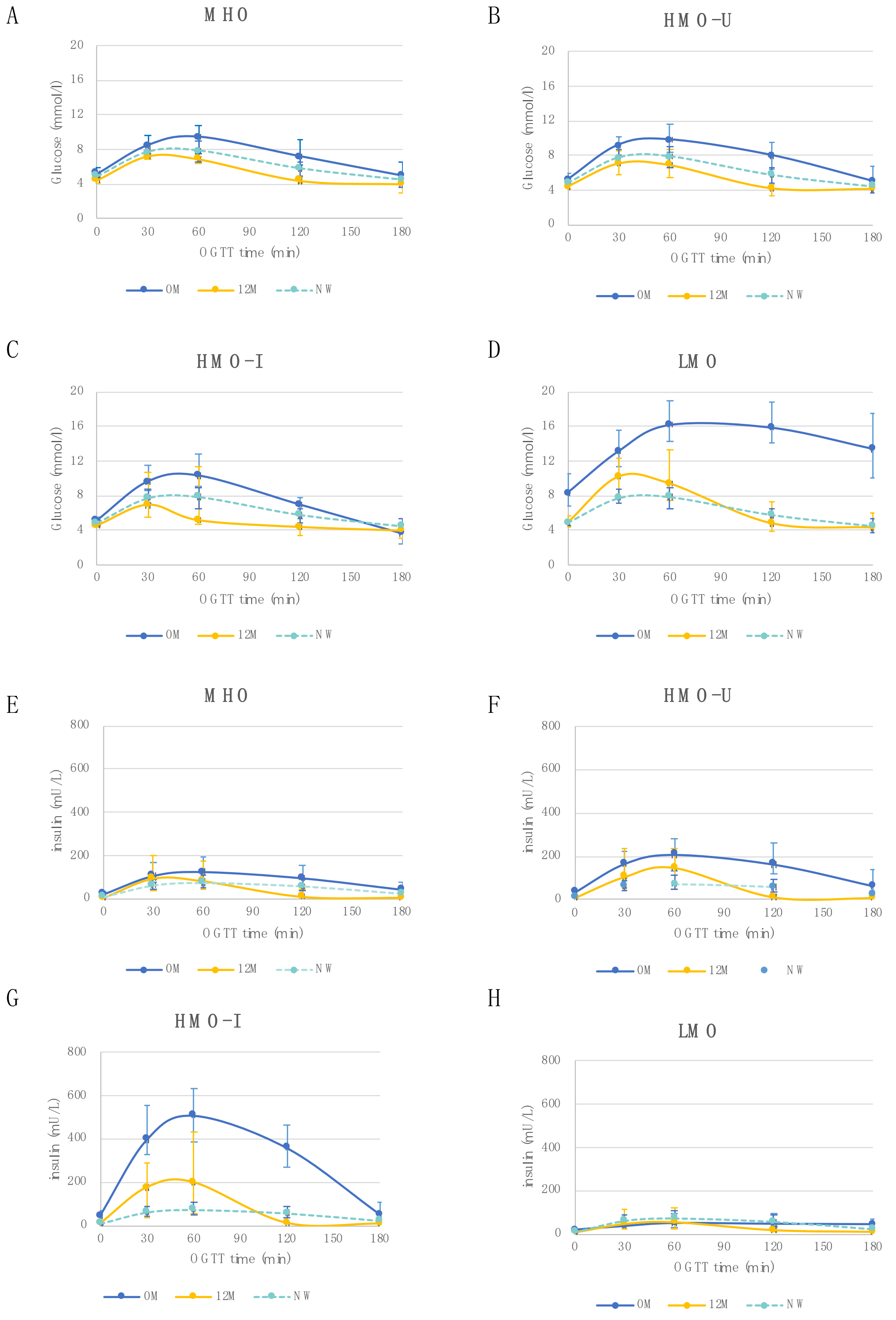

Figure S1 Comparison of different AIM subgroups of obesity and normal-weight controls for oral glucose tolerance test (OGTT) curves at baseline and 12-month post-surgery. A-D, OGTT for glucose; **E-H,** OGTT for insulin. Analysis are in only those patients having the 12-month follow-up visit. MHO, metabolic healthy obesity; HMO-I, hypermetabolic obesity hyperinsulinemia subtype; HMO-U, hypermetabolic obesity hyperuricemia subtype; LMO, hypometabolic obesity; NW, normal weight control; 0M, pre- surgery; 12M, 12-month post-surgery.

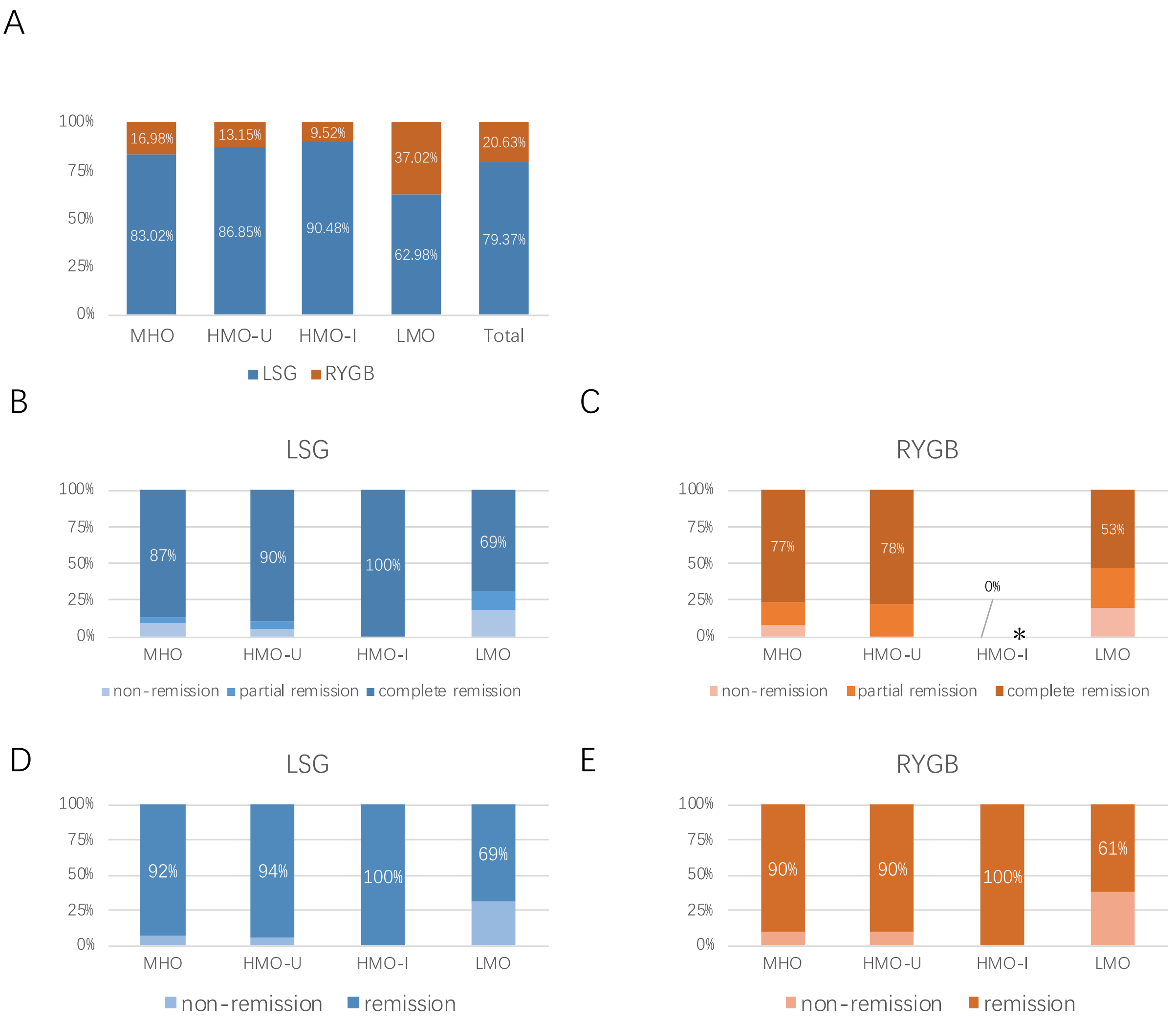

Figure S2 Comparison of different AIM subgroups of obesity for the remission rate of diabetes and hyperglycemia at 12-month post-surgery with respective to LGS and RYGB.

A, Proportion of LSG and RYBG with respective to each subgroup at baseline; **B, C,** Remission rate of diabetes at 12-month in LSG (B) and RYGB (C); **D, E,** Remission rate of hyperglycemia at 12-month in LSG (D) and RYGB (E). MHO, metabolic healthy obesity; HMO-I, hypermetabolic obesity hyperinsulinemia subtype; HMO-U, hypermetabolic obesity hyperuricemia subtype; LMO, hypometabolic obesity; LGS, Laparoscopic sleeve gastrectomy；RYGB, Roux-en-Y gastric bypass. * No patients with diabetes from HMO-I subgroup and performed with RYGB has the 12-month visit.

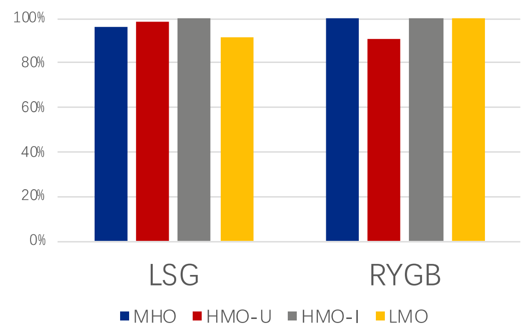

Figure S3 A. Comparison of different AIM subgroups of obesity for the remission rate of hyperinsulinemia in LGS and RYGB. MHO, metabolic healthy obesity; HMO-I, hypermetabolic obesity hyperinsulinemia subtype; HMO-U, hypermetabolic obesity hyperuricemia subtype; LMO, hypometabolic obesity; LGS, Laparoscopic sleeve gastrectomy；RYGB, Roux-en-Y gastric bypass.
